# Genetic variation and geographic differentiation in the marine triclad *Bdelloura candida* (Platyhelminthes, Tricladida, Maricola), ectocommensal on the American horseshoe crab *Limulus polyphemus*

**DOI:** 10.1007/s00227-017-3132-y

**Published:** 2017-04-20

**Authors:** Ana Riesgo, Emily A. Burke, Christopher Laumer, Gonzalo Giribet

**Affiliations:** 1 Department of Life Sciences (Invertebrate Division), The Natural History Museum of London, Cromwell Road, London, SW7 5BD UK; 2 Department of Organismic and Evolutionary Biology, Museum of Comparative Zoology, Harvard University, 26 Oxford Street, Cambridge, MA 02138 USA; 3 EMBL-European Bioinformatics Institute, Wellcome Trust Genome Campus, Hinxton, Cambridgeshire CB10 1SD UK; 4 Wellcome Trust Sanger Institute, Wellcome Trust Genome Campus, Hinxton, Cambridgeshire CB10 1SA UK

## Abstract

*Bdelloura candida* (Platyhelminthes, Tricladida, Maricola) is an ectocommensal symbiont on the American horseshoe crab *Limulus polyphemus*, living on the book gills and appendages, where it spends its entire life. Given its limited dispersal capabilities and its inability to live outside of the host, we hypothesized a genetic structure that parallels that of its host. We obtained 84 planarian individuals from 19 horseshoe crabs collected from 10 sites from Massachusetts to Florida. We amplified the mitochondrial 16S rRNA and the nuclear internal transcribed spacer 2 and conducted phylogeographic and population genetic analyses, which show a clear and strong genetic break between the populations in the Atlantic and the Gulf coasts. Among the Atlantic populations, two additional, weaker barriers located along Cape Hatteras and Cape Cod restrict gene flow. Even though previous studies have suggested that the populations of the host may be in decline, those of *B. candida* remain stable, and some even shows signatures of expansion. Our results indicate that the phylogeography of these marine ectocommensal triclads closely mirrors that of its *Limulus* host, and highlight the challenges to both host and symbiont to genetically connect populations across their distribution.

**Electronic supplementary material:**

The online version of this article (doi:10.1007/s00227-017-3132-y) contains supplementary material, which is available to authorized users.

## Introduction

The study of symbiosis is a growing field in biology, requiring integration of multiple disciplines (McFall-Ngai 2008). Symbiotic relationships among different phyla are often “loose”, especially among ectocommensal animals, which are commonly non-specific. However, many such ectocommensal relationships are known to be strictly specific, such as those of cycliophorans with their nephropid (clawed) lobsters (Funch and Kristensen 1995; Obst et al. 2006), where a faithful one-to-one species–host relationship exists, even in places where two host species coexist (Baker et al. 2007; Baker and Giribet 2007). In this case it is easy to appeal to the phenomenon of co-speciation, as each lobster host has a different ectocommensal species of *Symbion*, and these have never been found in non-nephropid hosts (it is thought that the American lobster can have up to three cryptic species of *Symbion* in the *S. americanus* complex, but these are not shared with any other host species). In general, symbiont and host share gene flow and their genetic structure is linked, the symbiont mirroring the genetic structure of the host (Blasco-Costa and Poulin 2013), although the global level of population genetic differentiation is usually lower in symbionts and parasites than in hosts (Mazé-Guilmo et al. 2016). However, species traits (e.g., size and life cycle) other than host genetic structure can have an effect in shaping symbiont structure, as shown in two recent meta-analyses of host-symbiont genetic structure (Blasco-Costa and Poulin 2013; Mazé-Guilmo et al. 2016).

A potentially similar case is that of the members of Bdellouridae, a family of marine planarians (Platyhelminthes, Tricladida, Maricola) and its host, the American horseshoe crab, *Limulus polyphemus* (Linnaeus, 1758). The members of the genus *Bdelloura* are characterized by the absence of eye lenses, a posterior adhesive caudal disk set off from the rest of the body, and numerous testes distributed throughout the body (Leidy 1851). The genus includes three described species, *Bdelloura candida* (Girard, 1850), *B. propinqua* Wheeler, 1894, and *B. wheeleri* Wilhelmi, 1909, of which *B. candida* is the most widespread and easily identified (Sluys 1989).

*Bdelloura candida* was described from Chelsea Beach, Massachusetts (Girard 1850). Their whitish-coloured, oval-shaped bodies are about 15 × 4 mm (sometimes up to 2 cm in length) while moving, according to Wilhelmi (1908). In his monograph, Sluys (1989) described *B. candida*’s characteristic undulated sides, large central pharynx, and broad caudal disk that changes shape depending on the state of contraction. *Bdelloura candida* lives ectocommensally on the walking appendages, carapace, and book gills of the Atlantic horseshoe crab. Despite relying heavily on their hosts for indirect nutrition, the feeding mechanisms and digestive structures of the commensal species of Bdellouridae do not differ significantly from those of their free-living relatives (Jennings 1977). *Bdelloura candida* can be found concomitantly with its host throughout its distribution range along the Gulf and Atlantic coasts of North America (Sekiguchi and Shuster 2009). As a direct developer (Sluys 1989), *B. candida* hatches from cocoons attached to the book gills of *L. polyphemus*, and it has, therefore, little capacity for dispersal. The adults cannot survive independently, living exclusively on its *L. polyphemus* host; thus, they must recolonize the same host or a nearby individual after *Limulus* moults, a process that probably relies on chemical signalling (Chevalier and Steinbach 1969). This strict association to horseshoe crabs makes *B. candida* an excellent model for phylogeographic research of symbiotic organisms.

The Atlantic horseshoe crab is distributed along the east coast of North America, from Maine to Florida, with additional populations in the eastern Gulf of Mexico and around the Yucatan peninsula (Anderson and Shuster 2003). Along this range, distinct populations of *L. polyphemus* are recognized (Shuster 1982; King et al. 2015), and their population sizes show a clear latitudinal gradient, the largest densities being found towards the middle of the range, in and surrounding Delaware Bay (e.g., Botton and Ropes 1987; Shuster 2003). However, many of these populations have suffered recent decline due to a diversity of anthropogenic factors (Faurby et al. 2010). Genetically, a marked structure exists among specimens north and south of northeastern Florida in mitochondrial DNA (Saunders et al. 1986a), allozymes (Selander et al. 1970), microsatellite nuclear markers (King et al. 2015), and morphology (Riska 1981), in what constitutes a well-documented marine biogeographic break (e.g., Avise 1992; Lee and Foighil 2004). This genetic structure most probably results from the territoriality of adults and the low dispersal capacity of the trilobite larva, which has been mainly studied in the Delaware estuary (Botton and Loveland 1987). Their transport as passive particles by the alongshore currents was estimated between 144 and 432 m per h, although they tend to remain in the close vicinity of the shoreline (Botton and Loveland 2003). Recapture data from *L. polyphemus* tag-and-release experiments provide a mean recovery distance of approximately 3–4 miles (Baptiste et al. 1957; Sokoloff 1978). Furthermore, many adults were shown to remain tightly associated with their particular estuary or local shoreline. This philopatry regarding reproductive and behavioural habits is also suggestive of a pattern of genetic structure.

Several authors have already postulated that genetic structure from some symbionts/parasites can mirror and even complement genetic data from their hosts (Nieberding and Olivieri 2007). Since genetic data are not available for *B. candida* to test whether its genetic structure mirrors that of *L. polyphemus*, we examined specimens from hosts at ten sites along the Atlantic and Gulf coasts of the USA, as we wanted to ascertain whether the commensal showed the signature of the host’s phylogeographic structure.

## Materials and methods

### Sample collection

A total of 84 specimens of *B. candida* were collected from 18 *L. polyphemus* individuals at ten sampling sites along the Atlantic and Gulf coasts (Table 1). Specimens were fixed in 96% ethanol and stored at −80 °C for long-term preservation. All host and planarian specimens are deposited in the Invertebrate Zoology collections in the Museum of Comparative Zoology (MCZ), Harvard University.

**Table 1:** Collection details for *Bdelloura candida* populations

Label	Acronym	Locality	Latitude	Longitude	*Limulus* hosts	Individuals
MAS-ORLEANS	MA1	Orleans	41.752°N	69.907°W	1	6
MAS-ANNISQUAM	MA2	Annisquam	42.655°N	70.682°W	1	1
CONNECTICUT	CT	Harvey’s Beach	41.274°N	72.395°W	3	14
DELAWARE	DE	Bower’s Beach	39.062°N	75.397°W	6	10
NORTH CAROLINA	NC	Morehead	34.698°N	76.676°W	1	5
SOUTH CAROLINA	SC	Isle of Palms	32.777°N	79.811°W	1	7
GEORGIA	GA	Cumberland Island	30.723°N	81.450°W	3	16
FLORIDA1	FL1	Crystal River	28.906°N	82.690°W	1	17
FLORIDA2	FL2	Saint Joseph Bay	29.766°N	85.404°W	1	3
FLORIDA3	FL3	Piney Island	30.020°N	84.385°W	1	7

### Molecular analyses

DNA was extracted using the DNEasy^®^ tissue kit (Qiagen, Valencia, CA, USA) following manufacturer’s instructions, with a slight modification in the incubation of the tissue in lysis buffer (overnight). We selected two markers, the mitochondrial 16S rRNA and the nuclear ribosomal internal transcribed spacer-2 region (ITS2). The 16S rRNA marker was amplified in 83 individuals with the primer pair 16Sa–16Sb (16Sa: 5′-CGC CTG TTT ATC AAA AAC AT-3′, 16Sb; 5′-CTC CGG TTT GAA CTC AGA TCA-3′; Xiong and Kocher 1991) to target a region of approximately 395 base pairs. The ITS2 marker was amplified in 52 individuals using universal primers 5.8SF (Carranza 1997) and 28S rev (Lôbo-Hajdu et al. 2003–2004; Duran et al. 2004). It is important to note here that we failed to sequence ITS2 from all individuals, in part because of an apparent indel-level variation within the many copies of the rRNA cistron, which may prevent correct amplification and/or direct Sanger sequencing of PCR products.

PCR reactions were conducted using 1 μL of template DNA, 18 μL molecular grade distilled water, 5 μL GoTaq ^®^ Buffer (Promega, Madison, WI, USA), 0.5 μL 10 mM dNTPs (New England Biolabs, Inc., Ipswich, MA, USA), 0.13 μL GoTaq^®^ Taq Polymerase (Promega, Madison, WI, USA), and 0.25 μL of each forward and reverse primers (10 mM). The PCR program for both markers included an initial denaturing step at 95 °C for 2 min, 35 cycles of denaturing at 95 °C for 30 s, annealing at 45 (for 16S rRNA) to 49 °C (for ITS2) for 30 s, and extension at 72 °C for 1 min, and then a final extension step at 72 °C for 1 min. The PCR products were vacuum cleaned and sequencing reactions were done using BigDye Terminator v3.1 (Life Technologies, Carlsbad, CA, USA) and a sequencing program with an initial denaturing step at 94 °C for 3 min, then 25 cycles of denaturing at 94 °C for 10 s, annealing at 50 °C for 5 s, and extension at 60 °C for 4 min. The purified PCR products were sequenced using the same primer pairs in a 3730xl DNA Analyzer (Life Technologies, Carlsbad, CA, USA) at the Harvard Center for Systems Biology.

Chromatograms were edited and assembled using Sequencher 5.0.1 (Gene Codes Corporation 1991–2011, Ann Arbor, MI, USA). Sequences were checked for contamination using BLAST (Altschul et al. 1997) on the NCBI website (http://ncbi.nlm.nih.gov). Accession numbers for 16S sequences in GenBank are KY499968-KY500051 (84 sequences) and those of ITS2 are KY500082-KY500133 (52 sequences).

### Sequence alignment

Sequence data were aligned using MAFFT version 7 (Katoh et al. 2002; Katoh and Standley 2013) on the developers’ online server (http://mafft.cbrc.jp/alignment/server/). For 16S rRNA, the L-INS-i strategy was used (Katoh et al. 2005). Because the DNA sequences were closely related, the scoring matrix for nucleotide sequences was set to 1PAM/*k* = 2. The offset value was set to 0.1 and the gap opening penalty (for all molecular markers) was 1.53. ITS-2 had similar settings, with the only change being the E-INS-i strategy (very slow, for sequences with multiple conserved domains and large gaps) (Katoh et al. 2005). Final length of the alignments after trimming the leading and trailing ends was 346 bp for 16S rRNA and 1248 bp for ITS2.

### Genetic diversity indices

Number of haplotypes and segregating sites, haplotype diversity, nucleotide diversity and mean number of pairwise differences were obtained in DNAsp 5.10.1 (Librado and Rozas 2009). Haplotype networks were estimated with TCS (Clement et al. 2000) implemented in PopART (http://popart.otago.ac.nz).

### Distribution of the genetic diversity

Genetic differentiation between populations was measured by computing pairwise *Ф*_ST_ statistics in ARLEQUIN v 3.5 (Excoffier and Lischer 2010), evaluating the corresponding *p* values by 10,000 permutations, which were then adjusted using a false discovery rate (Narum 2006). To test whether the data followed a pattern of isolation by distance, we performed a Mantel test in Genepop (Raymond and Rousset 1995) with 16,000 permutations, and converting the *Ф*_ST_ matrix to *Ф*_ST_/1 − *Ф*_ST_. In this case, only the 16S rRNA dataset, which contained all populations, was analysed. Geographical distances were calculated as the minimum distance in kilometres between sites. The effect of barriers in determining the genetic structure of *B. candida* populations was further evaluated using pairwise *Ф*_ST_ values and visualized with the software BARRIER v2.2 (Manni et al. 2004). BARRIER uses the matrix of geographical coordinates and links it with the corresponding distance matrix (*Ф*_ST_), and subsequently applies Monmonier’s distance algorithm to identify areas that hinder gene flow among sites, i.e., the zones where *Ф*_ST_ differences between pairs of sites are the largest.

Differentiation between groups of populations was assessed by conducting hierarchical analyses of molecular variance (AMOVA) using genetic distances, and testing their significance by running 16,000 permutations in ARLEQUIN. We grouped the populations in the following sets, based on results from BARRIER (see “Results” section): Gulf (FL1, FL2, FL3), Atlantic (GA, SC, NC, DE, CT, MA1, MA2), Group 1 (GA, SC, NC, DE, CT), Group 2 (GA, SC, NC), Group 3 (DE, CT), and Group 4 (MA1, MA2). We quantified three components of the genetic variance as follows: among groups [using three different combinations: (a) Gulf vs. Atlantic, (b) Gulf vs. Group 1 vs. Group 2, and (c) Gulf vs. Group 3 vs. Group 4], among populations within groups, and within populations. AMOVA analyses were conducted on the 16S rRNA and the ITS2 datasets separately.

### Demographic analyses

We performed the Tajima’s *D* test of neutrality to test for bottlenecks and population expansions (Tajima 1989). The history of effective population size was assessed through the pairwise mismatch distribution in ARLEQUIN, where populations in expansion show unimodal distributions while stationary populations show multimodal distributions. In addition, the neutrality Ramos-Onsins and Rozas’ *R*2 test was calculated for the four groups of populations obtained from Barrier [Gulf (FL1, FL2, FL3), Group 2 (GA, SC, NC), Group 3 (DE, CT), and Group 4 (MA1, MA2)] (Ramos-Onsins and Rozas 2002).

Given the high genetic divergence detected between the populations of Florida (“Gulf”) and those from the rest of the Eastern coast of the US (“Atlantic”) shown in “Results” section, we conducted a Bayesian analysis to assess migration rates between both regions in LAMARC v 2.1.9 (Kuhner 2006). Two initial runs were conducted to estimate the most likely priors for our dataset. Then, we used the Felsenstein’s 84 (F84) model of DNA sequence evolution and variation values of migration between 0 and 30 in both directions. The final run was based on four different replicates with 10 initial MCMC chains of 5000 generations each, a burning period of 500, followed by 2 final MCMC chains of 40,000 generations each with a burning period of 1000. Two simultaneous heating searches (1 and 1.5) were performed per replicate. To test convergence of the chains and confirm the existence of at least 200 independent simulations (effective sample size—ESS) for each parameter, results were summarised in Tracer v 1.5 (http://beast.bio.ed.ac.uk/Tracer). Migration rates (Mt) were expressed as the number of migrants per generation Mt = *n*/*µ*, where *n* is the immigration rate per generation, and *µ* the substitution rate.

## Results

### Genetic diversity and variation in *Bdelloura candida*

**Fig. 1:**
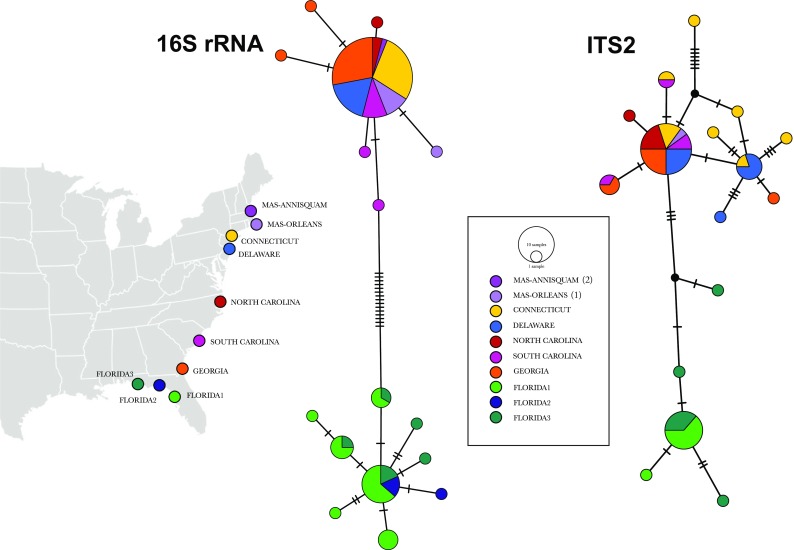
Map of sampling sites for *Bdelloura candida* and haplotype networks reconstructed with PopART for the 16S rRNA and ITS2 markers. *Crossing lines* between haplotypes indicate mutational steps

All values of genetic diversity are detailed in Table 2. Surprisingly, for most populations, haplotype diversity was higher for the ITS2 than for 16S rRNA, given that less individuals were sequenced for ITS2 than for 16S rRNA. We found 11 private haplotypes for each marker (68.75% of total haplotypes for 16S rRNA and 55% for ITS2). Values of nucleotide diversity were generally low (<0.0055) except for FL3 and NC (for the 16S rRNA dataset). The haplotype networks of both markers (Fig. 1) showed a clear separation between the populations along the Gulf of Mexico (FL1, FL2, and FL3) and the haplotypes found in the populations of the East Atlantic coast (MA1, MA2, CT, DE, NC, SC, and GA), connected by 15 mutational steps in the 16S rRNA network and three in ITS2 (Fig. 1). There were no shared haplotypes between the populations of the Gulf of Mexico and those of the Atlantic coast (Fig. 1). The 16S rRNA haplotype network contained a single common haplotype distributed in all populations of the Atlantic coast and six private haplotypes (Fig. 1), while in the Gulf coast, we found one haplotype distributed in ten individuals, three more haplotypes in three individuals, and five private haplotypes (Fig. 1). The ITS2 haplotype network showed two main haplotypes in the Atlantic coast without a clear geographic pattern and seven private haplotypes (Fig. 1), while in the Gulf we found one main haplotype and three private haplotypes (Fig. 1).

**Table 2:** Genetic diversity and demographic estimates for *Bdelloura candida* populations

Population	16S rRNA						ITS2					
	*N*	*S*	*h*	Hd	π	Tajima’s *D*	*N*	*S*	*h*	Hd	π	Tajima’s *D*
MA1	6	1	2	0.3333	0.00145	−0.93302	–	–	–	–	–	–
MA2	1	–	–	–	–	–	–	–	–	–	–	–
CT	13	0	1	0	0	–	9	13	8	0.97222	0.00307	−1.12439
DE	9	0	1	0	0	–	10	5	5	0.8444	0.00134	−0.32944
NC	3	3	2	0.6667	0.0087	–	5	2	3	0.8	0.00083	0.24314
SC	7	2	3	0.52381	0.00248	−0.93302	4	3	4	1	0.00138	0.16766
GA	16	2	3	0.24167	0.0019	−1.49796	8	4	5	0.78571	0.00112	−0.52474
FL1	17	6	6	0.79412	0.0055	−0.96394	8	2	3	0.67857	0.00068	−0.62573
FL2	3	1	2	0.66667	0.0029	0	–	–	–	–	–	–
FL3	7	6	5	0.85714	0.00745	−1.52412	7	6	5	0.85714	0.00181	−0.53627
Total	81	28	16	0.62778	0.03137	0.43826	51	27	20	0.90588	0.00322	−1.16766

*N* number of individuals, *S* number of segregating sites *h* number of haplotypes, Hd haplotype diversity, *π* nucleotide diversity

**Fig. 2:**
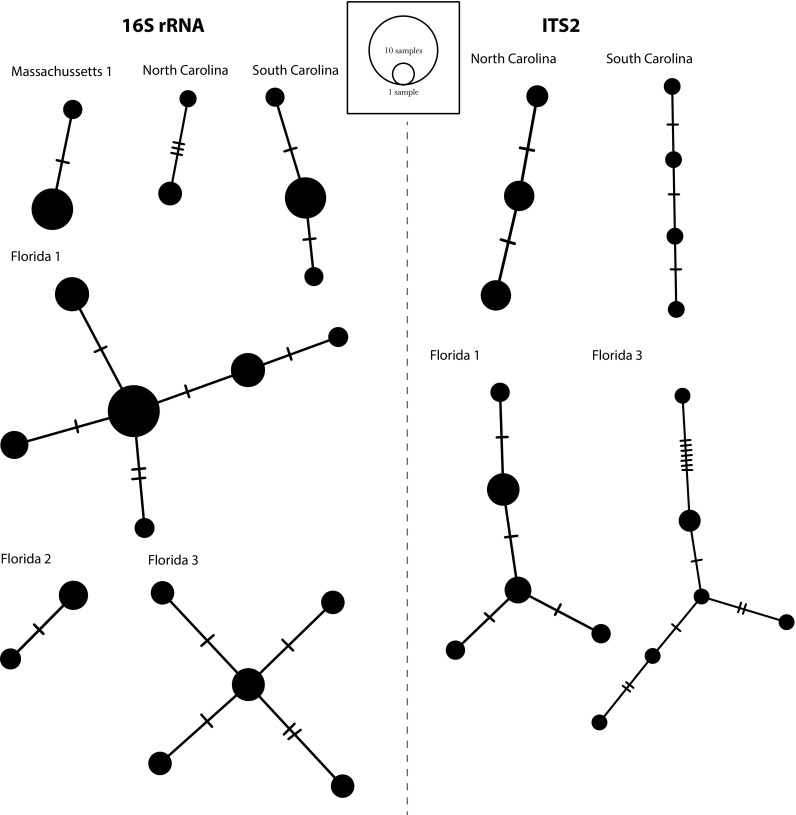
Haplotypic networks for each population of *Bdelloura candida* from only one sampled individual host, reconstructed with PopART for the 16S rRNA and ITS2 markers

**Fig. 3:**
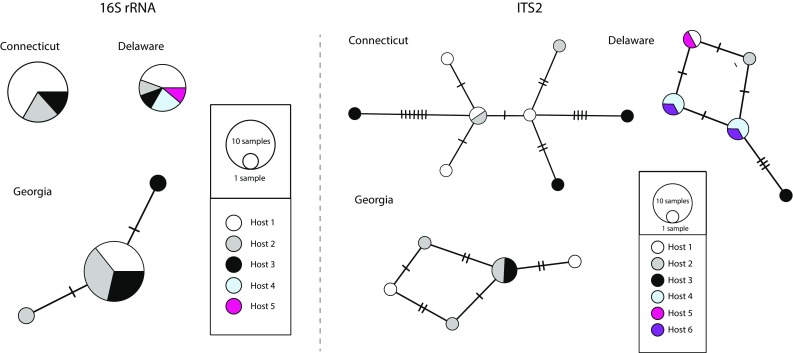
Haplotype networks for each population of *Bdelloura candida* collected from more than one individual host reconstructed with PopART for the 16S rRNA and ITS2 markers. Note that *colours* do not indicate identical haplotypes within populations

When the populations were analysed separately to evaluate the genetic diversity contained per host, we observed that not all individuals of *B. candida* collected from the same host were genetically identical (Fig. 2). In turn, 2–6 haplotypes were found within the same horseshoe crab host for both markers (Figs. 2, 3). When several hosts were collected, in some cases only one haplotype was recovered for *B. candida* in all hosts, for instance in Connecticut and Delaware for 16S rRNA (Fig. 3), while in most cases more than one *Bdelloura* haplotypes were collected in each host, especially for ITS2 (Fig. 3).

The *Ф*_ST_ values for both markers showed mainly significant differences between pairwise comparisons among populations from the Gulf of Mexico and those from the Atlantic coast (Table 3). We found that both the Atlantic coast and the Gulf populations were panmictic (Table 3).

**Table 3:** Φ_ST_ values for 16S rRNA and ITS2

16S rRNA										
	MA1	MA2	CT	DE	NC	SC	GA	FL1	FL2	FL3
MA1	0.0000									
MA2	0.0000	0.0000								
CT	0.13907	0.0000	0.0000							
DE	0.07216	0.0000	0.0000	0.0000						
NC	0.0000	0.0000	0.4902	0.37931	0.0000					
SC	0.0000	0.0000	0.19404	0.1272	0.0000	0.0000				
GA	0.0000	0.0000	0.01727	0.0000	0.07438	0.01268	0.0000			
FL1	**0.37477**	0.20588	**0.56753**	**0.52336**	**0.24357**	**0.31362**	**0.47731**	0.0000		
FL2	**0.55372**	0.33333	**0.88841**	**0.84874**	0.33333	**0.42882**	**0.67536**	0.0000	0.0000	
FL3	**0.39139**	**0.14286**	**0.67788**	**0.61468**	0.21192	**0.30952**	**0.53299**	0.0000	0.0000	0.0000

ITS2								
	MA2	CT	DE	NC	SC	GA	FL1	FL3
MA2	0.0000							
CT	0.0000	0.0000						
DE	0.0000	0.0000	0.0000					
NC	0.0000	0.0000	0.0000	0.0000				
SC	0.0000	0.0000	0.0000	0.0000	0.0000			
GA	0.0000	0.0000	0.01104	0.0000	0.0000	0.0000		
FL1	0.32143	**0.17113**	**0.23438**	**0.26880**	0.19086	**0.26786**	0.0000	
FL3	0.14286	**0.08284**	**0.14964**	**0.16897**	0.08117	**0.17963**	0.00225	0.0000

**Fig. 4:**
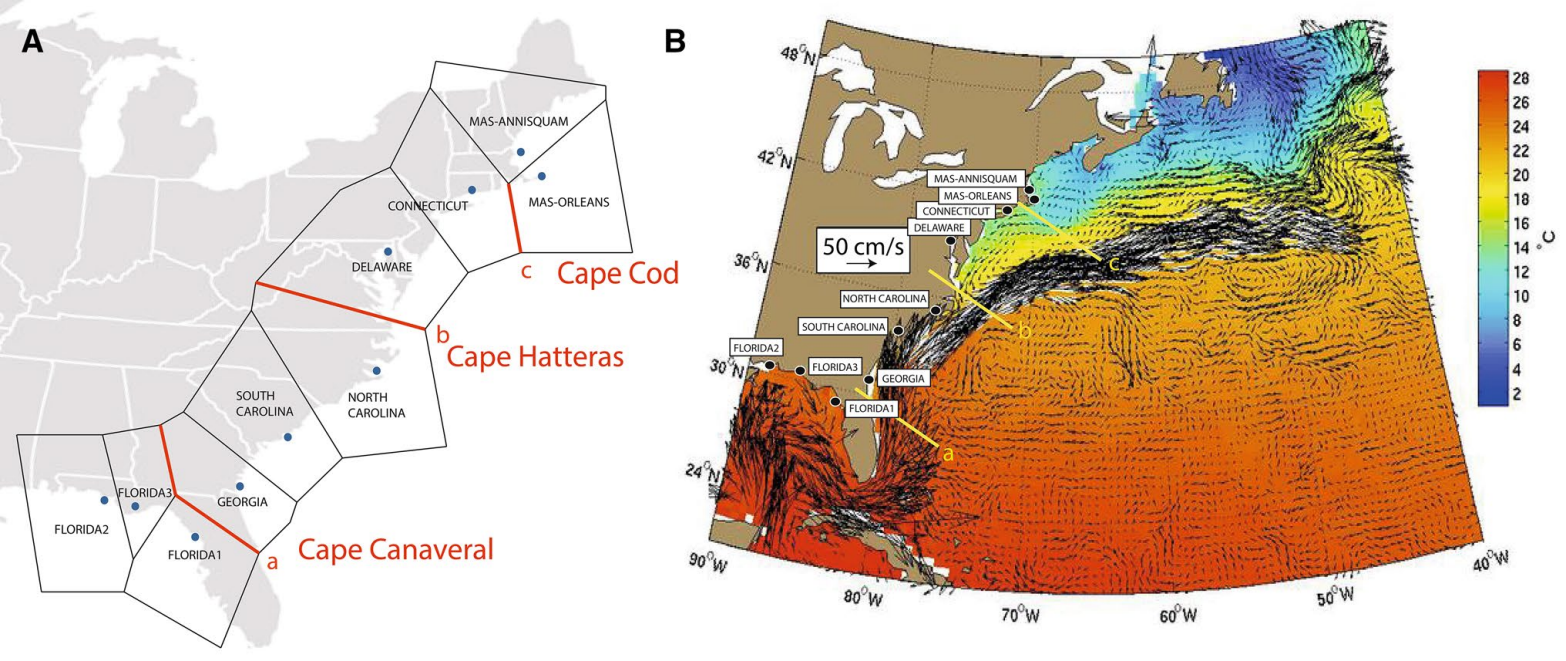
Location of major genetic breaks and oceanographic fronts in *Bdelloura candida*. **A** Geographic location of three major genetic breaks in the 16S rRNA dataset ranked in order of importance from *a* to *c*. Polygons indicate Delaunay triangulations obtained from Voronoï tessellations. **B** Reconstruction of seasonal averages for major currents using Mariano Global Surface Velocity Analysis (MGSVA) in the North Atlantic coast of the United States (from Gyory, J. Mariano, A.J., Ryan, E.H. The Gulf Stream. Ocean Surface Currents. http://oceancurrents.rsmas.miami.edu/atlantic/gulf-stream.html)

Using the matrix of *Ф*_ST_ values for 16S rRNA and the geographic coordinates, BARRIER located the three a priori selected barriers in Cape Canaveral, Cape Hatteras and Cape Cod (Fig. 4a, b), grouping the populations in four groups (Gulf, Groups 1, 3, and 4). All AMOVA analyses for both markers showed significant differences among groups and among individuals within populations (Tables 4, 5). However, the percentage of variation obtained when analysing the differences among groups was highest when considering four groups (Gulf vs. Group 1 vs. Group 3 vs. Group 4) for 16S rRNA (Table 4) and when analysing three groups (Gulf vs. Group 1 vs. Group 2) for ITS2 (Table 5). This genetic differentiation could be attributed to a pattern of isolation by distance (Supplementary Figure 1; *p* = 0.005 for 16S rRNA and *p* = 0.0105 for ITS2).

**Table 4:** Results of the AMOVA analyses for each of the combinations of populations (groups) for the 16S rRNA

Source of variation	*df*	Sum of squares	Variance components	Percentage of variation	Fixation indices	*p* value
16S Atlantic versus Gulf						
Among groups	1	9.4780	0.25818	56.98	0.56976	0.00914
Among populations within groups	8	1.2540	−0.00608	−1.34	−0.03119	0.55664
Within populations	72	14.4750	0.20104	44.37	0.55634	0.00000
Total	81	25.2070	0.45314			
16S Gulf versus Group 1 versus Group 2						
Among groups	2	9.5520	0.2078	51.37	0.5137	0.00065
Among populations within groups	7	1.1810	−0.0044	−1.07	−0.0221	0.5361
Within populations	72	14.4750	0.2010	44.37	0.5030	0.0000
Total	81	25.2070	0.4045			
16S Gulf versus Group 1 versus Group 3 versus Group 4						
Among groups	3	265.3330	4.5273	93.35	0.9335	0.0064
Among populations within groups	6	2.3490	0.0120	0.25	0.0373	0.2627
Within populations	72	22.3500	0.3104	6.40	0.9360	0.0000
Total	81	290.2320	4.8498			

Groups: Gulf (FL1, FL2, FL3), Atlantic (GA, SC, NC, DE, CT, MA1, MA2), Group 2 (GA, SC, NC), Group 3 (DE, CT), and Group 4 (MA1, MA2)

*df* degrees of freedom

**Table 5:** Results of the AMOVA analyses for each of the combinations of populations (groups) for the ITS2

Source of variation	*df*	Sum of squares	Variance components	Percentage of variation	Fixation indices	*p* value
ITS2 Atlantic versus Gulf						
Among groups	1	2.5200	0.1039	20.42	0.5137	0.00065
Among populations within groups	6	1.9560	−0.0154	−3.02	−0.0221	0.5361
Within populations	44	18.4850	0.4201	82.60	0.5030	0.0000
Total	51	22.9620	0.5086			
ITS2 Gulf versus Group 1 versus Group 2						
Among groups	2	51.0910	2.1503	68.92	0.6892	0.0069
Among populations within groups	5	6.0810	0.0404	1.30	0.0417	0.1334
Within populations	44	40.8850	0.9292	29.78	0.7022	0.0000
Total	51	98.0580	3.1199			
ITS2 Gulf versus Group 1 versus Group 3 versus Group 4						
Among groups	3	53.8870	1.4609	61.52	0.6152	0.00579
Among populations within groups	4	3.2850	−0.0156	−0.65	−0.0170	0.4668
Within populations	44	40.8850	0.9292	39.13	0.6087	0.0000
Total	51	98.0580	2.3646			

Groups: Gulf (FL1, FL3), Atlantic (GA, SC, NC, DE, CT, MA2), Group 2 (GA, SC, NC), Group 3 (DE, CT), and Group 4 (MA2)

*df* degrees of freedom

### Demographic analyses

**Fig. 5:**
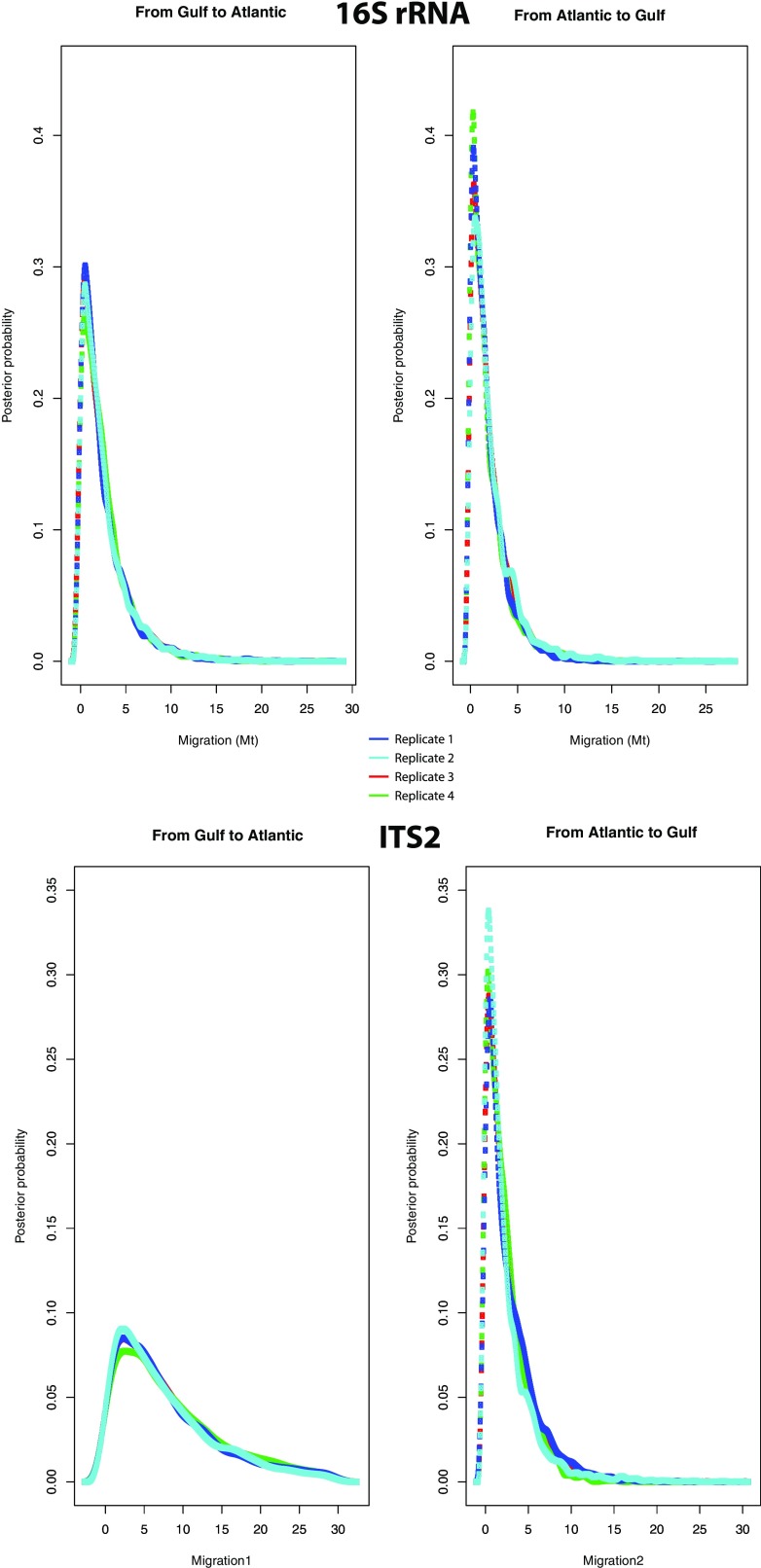
Graphical representation of the results for the migration analysis between Atlantic and Gulf populations in *Bdelloura candida* using the 16S rRNA and ITS2 markers. The *four graphs* show the five replicates used in LAMARC to analyse the migration rate (Mt) from the Gulf to the Atlantic (*left graphs*) and from the Atlantic to the Gulf of Mexico (*right graphs*)

Neutrality tests were conducted for those populations for which more than three individuals were available. The Tajima’s *D* statistics were not significant in any of the populations, indicating no evidence of selection (Table 2), and the mismatch distribution plots suggested population expansions in almost all populations analysed (Supplementary Figure 2). When grouping the populations into four groups (Gulf, Group 2, Group 3 and Group 4), the Ramos-Onsins & Rozas’ *R*2 test was significant for the Gulf populations for both markers and also for Group 3 (DE, CT) for ITS2 (Table 6), and therefore, a null hypothesis of neutral evolution under a constant population size can be rejected. However, Tajima’s *D* was significant only in Group 2 (GA, SC, NC) for the 16S rRNA marker (Table 6). LAMARC did not show clear patterns of migration in any direction (Fig. 5) between the two main groups of populations (Gulf to Atlantic or Atlantic to Gulf).

**Table 6:** Demographic estimates for the four groups of populations shown in Fig. 2 [Gulf (FL1, FL2, FL3), Group 2 (GA, SC, NC), Group 3 (DE, CT), and Group 4 (MA1, MA2)]

Population	*R*2	95% CI	Tajima’s *D*
16S rRNA			
Gulf	**0.0743**	(0.07667, 0.22667)	−1.78821
Group 2	0.0934	(0.07385, 0.25846)	−2.16252
Group 3	–	–	–
Group 4	0.3499	(0.0000, 0.71429)	−1.00623
ITS2			
Gulf	**0.0908**	(0.10070, 0.24944)	−1.42743
Group 2	0.1139	(0.09762, 0.25368)	−0.90835
Group 3	**0.0818**	(0.08496, 0.22473)	−1.65681
Group 4	–	–	–

Significant values are shown in bold letters. Absence of values reflect populations without polymorphisms

*R2* Ramos–Onsins statistic, *CI* confidence interval

## Discussion

### Genetic diversity estimates for *Bdelloura candida*

The low values of nucleotide diversity (*π*) of *B. candida* were similar to those of other triclads (e.g., Sunnucks et al. 2006; Álvarez-Presas et al. 2012), although these are terrestrial free-living species, which makes the comparisons less relevant given the peculiarities of their habitats. The Ramos-Onsins & Rozas’ *R*2 and Tajima’s *D* neutrality tests were mostly non-significant, which indicates a certain level of stability, although sample sizes were small, and therefore, conclusions from Tajima’s *D* test should be taken with caution. However, the Gulf region did show evidence of population expansion for both markers, as well as the group comprised by North Carolina, South Carolina and Georgia for 16S rRNA when using Ramos-Onsins & Rozas *R*2 test, which is robust to small sample sizes. Signatures of population expansion were also detected in the group comprised by Delaware and Connecticut for ITS2. However, such values could also be the consequence of purifying selection acting on the mitochondrial or nuclear genomes. Even if populations of *B. candida* were not in expansion but remained stable, these results are intriguing since the host *L. polyphemus* has been in decline over the recent decades (Gómez-Aguirre 1993; Faurby et al. 2010), and one could expect a similar trend in the viability of the populations of both the symbiont and the host. Perhaps, since each host can maintain up to dozens of bdellourid specimens, a decline in the horseshoe crabs should actually reach a certain threshold before affecting the genetic diversity in the symbionts.

Given the life cycle of *B. candida*, where adults have to change host during or after moulting, and therefore, a random pool of haplotypes might be colonizing the horseshoe crabs each time, low levels of clonality were expected. Indeed, in some cases we found high haplotypic diversity within the same individual host, with 2–6 haplotypes appearing concomitantly for both markers.

### Phylogeographic patterns: genetic breaks and geographic barriers

The genetic structure of *B. candida* along the Atlantic coast of the United States mirrors that of its host, *L. polyphemus*, as usually occurs in host–symbiont relationships (Mazé-Guilmo et al. 2016). In our results, the correlation of geographic distance and genetic differentiation in both genetic markers indicates a pattern of isolation by distance, also recognized in *L. polyphemus* populations (King et al. 2015), which is compatible with certain level of genetic structuring along the Atlantic coast of North America. The results of the haplotype networks, *Ф*_ST_, and AMOVA analyses indicate a significant genetic break between the Atlantic and Gulf coast populations of *B. candida*, which the software BARRIER locates primarily along the Atlantic coast of Florida. Geographic and ecological barriers may influence the dispersal capabilities of an organism, as well as its biological features limiting potential for long-distance dispersal and playing a role in genetic and geographic variation of a species (Gawarkiewicz et al. 2007). In this sense, it seems that the Florida peninsula represents a strong barrier to the already-limited dispersal capacity of *B. candida*.

Several authors have also previously found a sharp genetic break within the continuously distributed Atlantic and Gulf coast populations of *L. polyphemus* (Selander et al. 1970; Riska 1981; Saunders et al. 1986b; King et al. 2015), which parallels our analyses of *B. candida.* Riska (1981) found that populations of *L. polyphemus* from Cape Cod to Mexico showed unusually large morphological differences, exhibiting a north–south cline associated with the location of particular spines on the body and the length of the doublure anterior to the mesial spine. The major genetic break for *L. polyphemus* occurs in Northern Florida, approximately in the Indian River region, a region long-recognized as a transition zone between temperate and tropical marine ecosystems and fauna that exhibit analogous phylogeographic patterns (Briggs 1974). The Indian River region is known for its peculiarities, showing highly variable salinity measurements depending on distance from the inlets and the magnitude of freshwater inputs (Ehlinger and Tankersley 2004), which indeed hampers gene flow in *L. polyphemus* (King et al. 2015) and potentially in its symbionts.

Another physical barrier located in Florida, potentially responsible for the genetic structure of *B. candida*, is surface temperature and movement of water masses, since the limits of the *L. polyphemus* distribution are mostly determined by temperature (Sekiguchi and Shuster 2009). The temperature clines of approximately 24–27 and 27–30 °C surrounding southern Florida are visible in both day and night observations, and may represent a significant water mass boundary that isolates the Atlantic Ocean and Gulf of Mexico populations of ectocommensal marine triclads. In addition to physical barriers, ecological barriers in this region may also help explain the genetic break between the Atlantic and Gulf populations. The rapid urbanization of the Atlantic coast of Florida has severely impacted the beaches (Halpern et al. 2008), destroying the available habitats for *L. polyphemus* (Walls et al. 2002; Shuster 2003; Shuster and Sekiguchi 2009; Faurby et al. 2010).

The divergence in the genetic structure of the populations of *B. candida* from the Gulf of Mexico and the Atlantic coast is also maintained due to the scarce migration detected among the regions in both directions, even though the Shelf break frontal jet and the Gulf Stream may sustain some level of gene flow in this area for other organisms (Hare and Avise 1998; Gawarkiewicz et al. 2007). The Gulf Stream allows genetic connectivity across major barriers for some species (Gawarkiewicz et al. 2007), although in some instances it is insufficient and leads to lineage diversification (Boehm et al. 2013). Given that *B. candida* cannot survive outside its host for long periods and lacks a larval phase, gene flow between divergent regions is presumably only allowed when the adult host migrates. In this sense, the strong territoriality of the horseshoe crabs (Botton and Loveland 2003), together with the well-established oceanographic and physical barriers, severely restrict the genetic connectivity of *B. candida*.

Two additional barriers to gene flow in *B. candida* were also located along the US East coast between North Carolina and Virginia and between Rhode Island and Massachusetts (Fig. 2). These two barriers correspond well with the fronts created by the Gulf Stream flowing to Cape Hatteras, and Cape Cod isolating the colder waters of the Gulf of Maine. Indeed, around Cape Hatteras, there is a strong eastward flow of the Gulf Stream, which could constitute an effective barrier to gene flow (Fig. 2b). In addition, time/averaged flow plots of surface ocean currents (Fig. 4b) shows recirculation of waters along the coasts between Virginia and Massachusetts (also seen in Maximenko et al. 2009). Such fronts have shown strong influence on the pattern of genetic differentiation between the populations of the Atlantic coast of both *B. candida* and *L. polyphemus* (Selander et al. 1970; Saunders et al. 1986a; King et al. 2015), as well as in other marine organisms (see reviews by Avise 1992, 1996). However, the genetic structure of *B. candida* is not entirely mirrored by that of *L. polyphemus*, which showed great similarities in the genetic patterns of horseshoe crab populations of Connecticut, Delaware and North Carolina (King et al. 2015), while in *B. candida* North Carolina groups with South Carolina and Georgia (Fig. 4). Denser sampling along this region may serve to refine these barriers.

### Climate-related factors influencing genetic structure in *Bdelloura candida*

*Bdelloura candida*’s patterns of genetic variation can be influenced by other factors besides present-day physical and oceanographic barriers, among which climate may be one of the strongest factors. It is likely that the conditions of the last glacial maximum dramatically affected *L. polyphemus* population sizes and that these populations gradually expanded their range northward when the ice receded, but their genetic diversity had been severely compromised (Faurby et al. 2010), at least in the northern range of its distribution. Genetic signatures of a recent post-glacial population expansion in *B. candida* can be hinted in the 16S rRNA haplotype network (Fig. 1), with a characteristic star-like structure showing private haplotypes radiating from a single dominant haplotype, although the small sampling size makes this conclusion tentative.

In addition to the end of the last ice age, major climactic oscillations have contributed to shifting ranges of species in the North Atlantic (e.g., Maggs et al. 2008). These changes in climate affect the genetic structure and diversity of populations, and even entire species (Faurby et al. 2010). These post-Pleistocene climate fluctuations have also influenced physical and ecological properties of the east coast of North America, potentially causing both ancient divergence and some of the geographic barriers that still exist today, e.g., present-day coastal currents and ocean circulation patterns, and glacial moraines like Long Island and Cape Cod (Faurby et al. 2010).

## Conclusion

There is a strong agreement in all our molecular analyses indicating a major genetic break between the Gulf and Atlantic coast populations of *B. candida*, a break that has been identified in many other marine organisms (Avise 1992). In this sense, the phylogeography of these marine parasitic triclads closely mirrors that of its host. However, future studies examining the genetic diversity and geographic differentiation of *B. candida*, or marine triclads in general, are needed to elucidate the exact location of the boundary zone between the Atlantic and Gulf coast populations. Southeastern Florida, in particular, is poorly sampled for both *L. polyphemus* and *B. candida*, as animals are much more difficult to find in the southern ranges of their distribution. In this context, future sampling in the disjunct populations known from the Yucatan peninsula should also help to refine southerly geographic barriers in this species. In addition, given the strong nature of the major barrier isolating the populations from the Gulf of Mexico from those of the Atlantic coast of the United States, morphological analyses of *B. candida* are needed to help elucidate potential speciation processes that are also currently hinted in the host.

## Electronic supplementary material

Below is the link to the electronic supplementary material.

Supplementary material 1 (PDF 181 kb)

Supplementary material 2 (PDF 297 kb)
