# Supplementary material for: Genetic variation and geographic differentiation in the marine triclad Bdelloura candida (Platyhelminthes, Tricladida, Maricola), ectocommensal on the American horseshoe crab Limulus polyphemus
Source: Mar Biol. 2017 Apr 20;164(5):111. doi: 10.1007/s00227-017-3132-y (PMC5397438; doi:10.1007/s00227-017-3132-y)
Supplement: Supplementary file 1 — Supplementary material 1 (PDF 181 kb) [file 227_2017_3132_MOESM1_ESM.pdf]

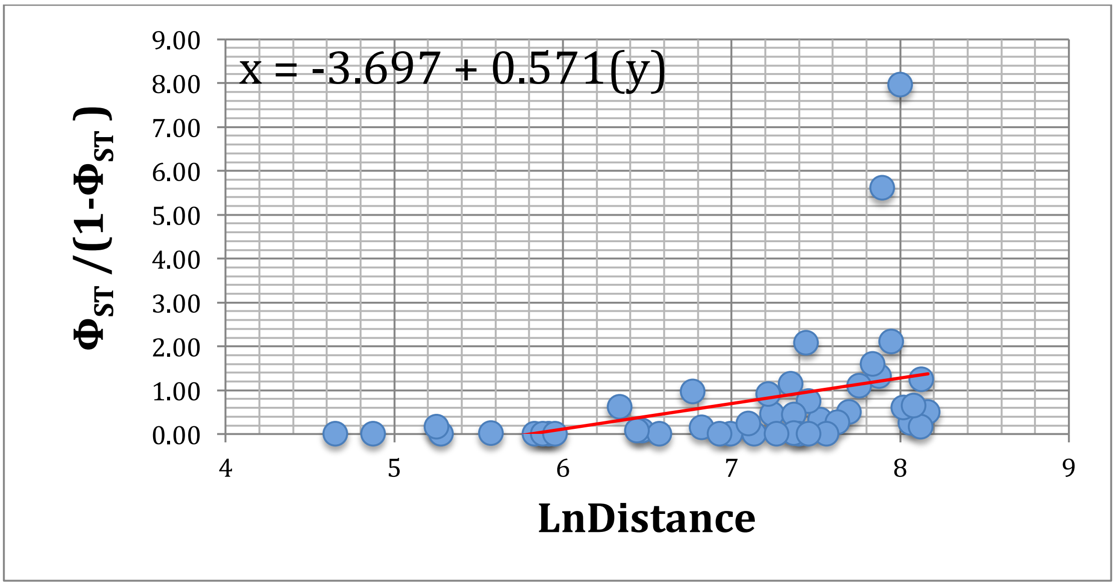

Supplementary Figure 1A. Plot of the logarithmic distances against the Φ_{ST} /(1-Φ_{ST}) values of genetic differentiation for the 16S rRNA marker.

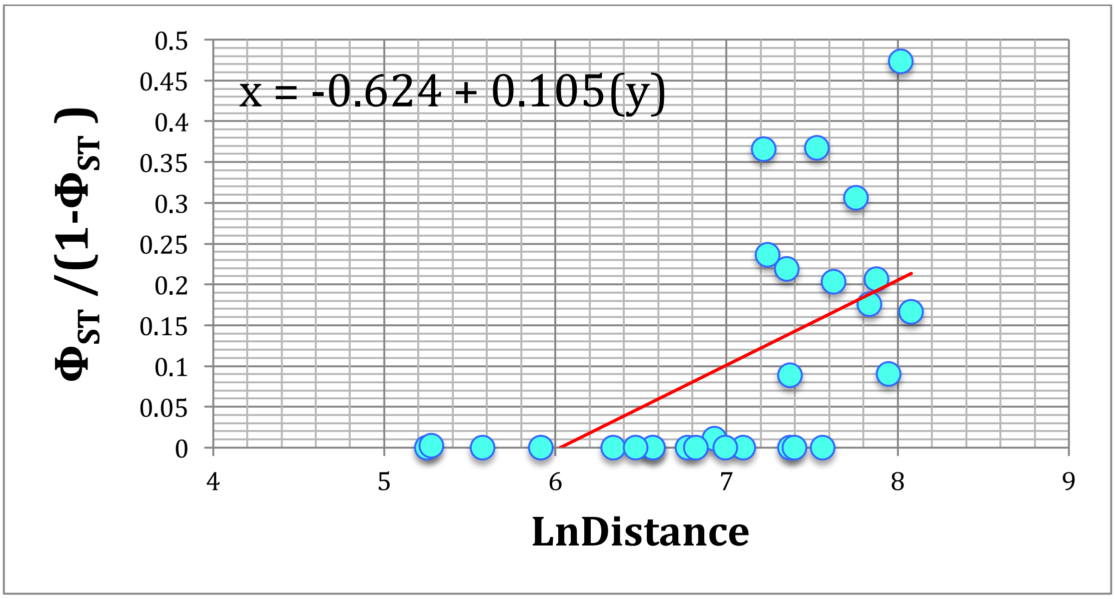

Supplementary Figure 1B. Plot of the logarithmic distances against the Φ_{ST} /(1-Φ_{ST}) values of genetic differentiation for the ITS2 marker.
