# Supplementary figures and images for: Genetic variation and geographic differentiation in the marine triclad Bdelloura candida (Platyhelminthes, Tricladida, Maricola), ectocommensal on the American horseshoe crab Limulus polyphemus
Source: Mar Biol. 2017 Apr 20;164(5):111. doi: 10.1007/s00227-017-3132-y (PMC5397438; doi:10.1007/s00227-017-3132-y)

16S rRNA

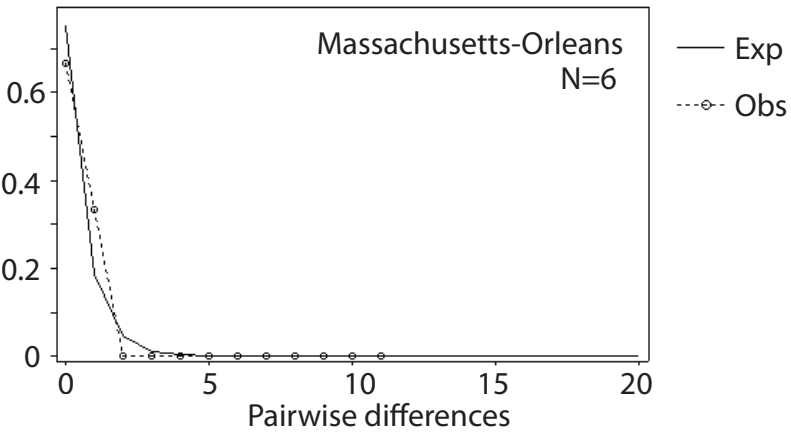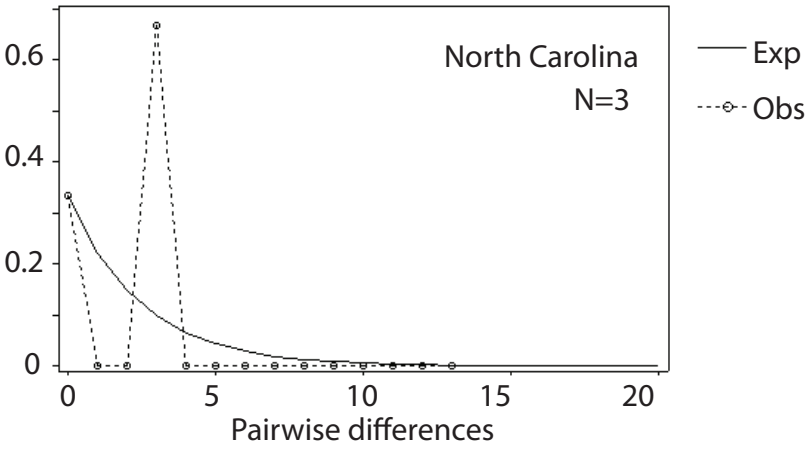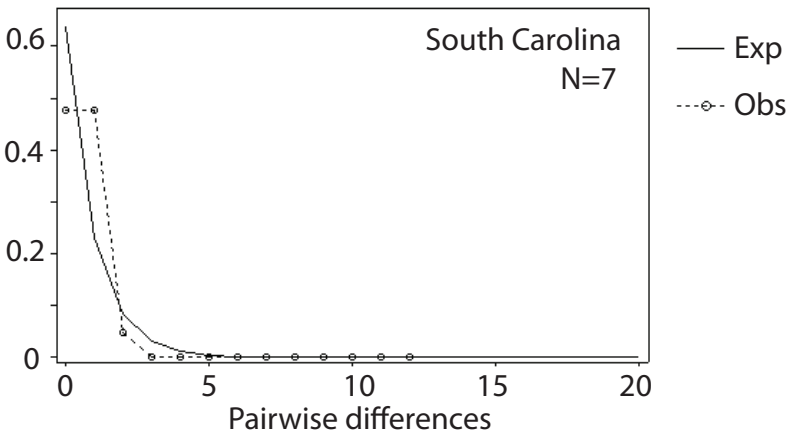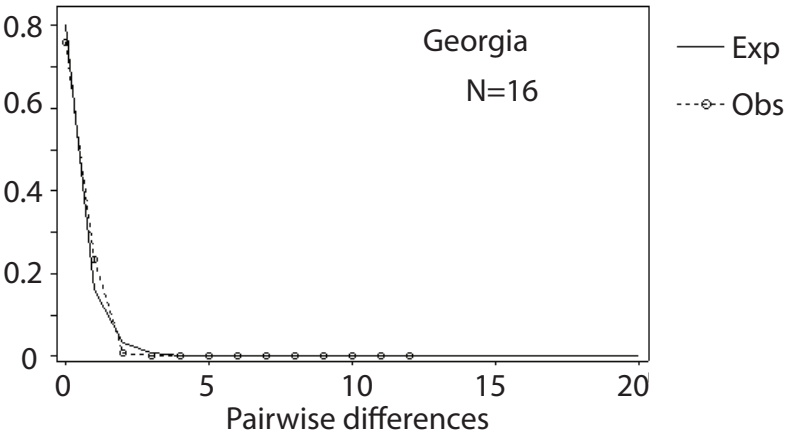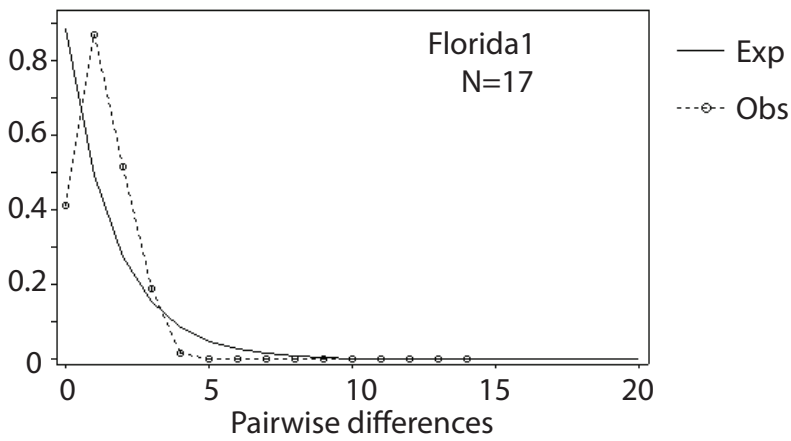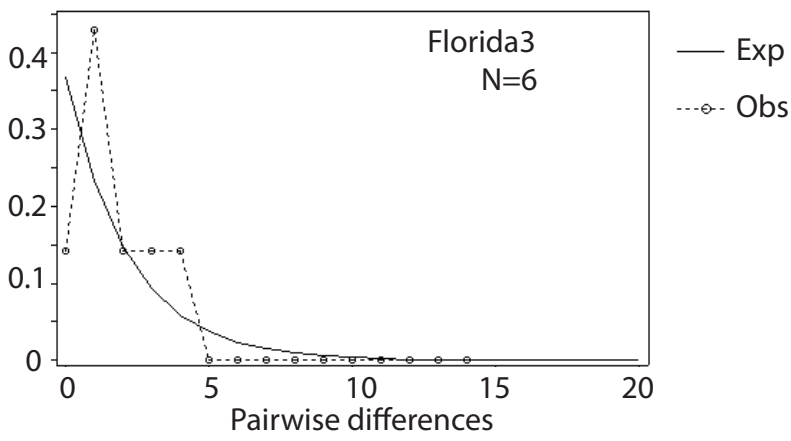

ITS2

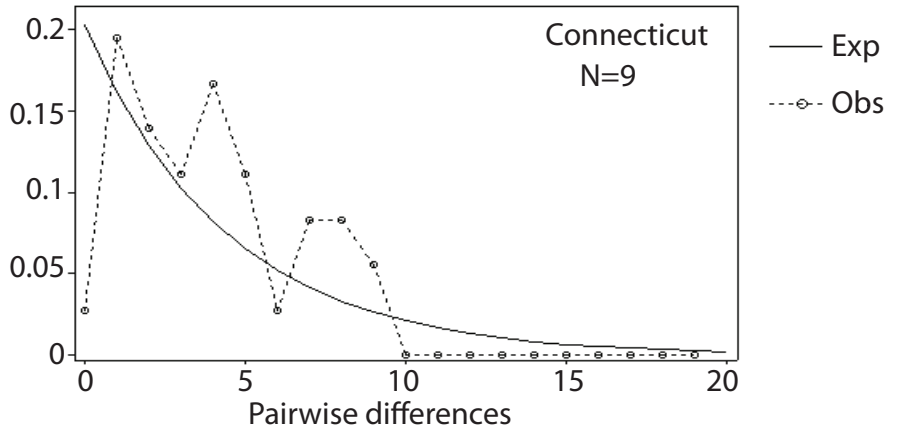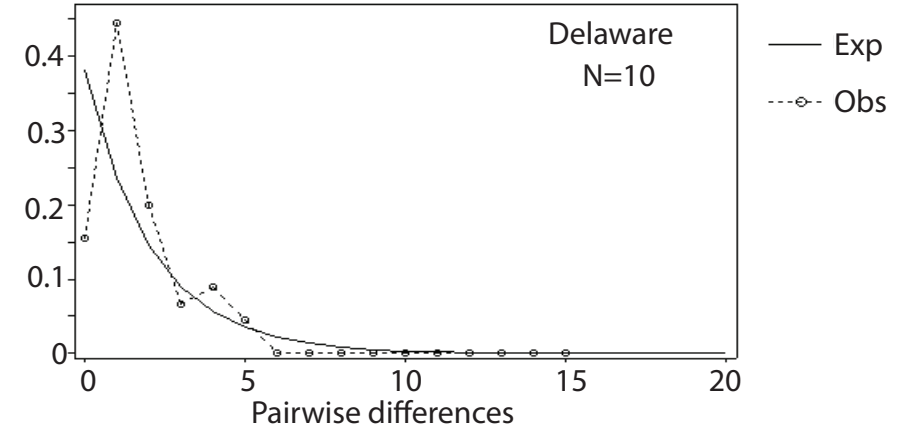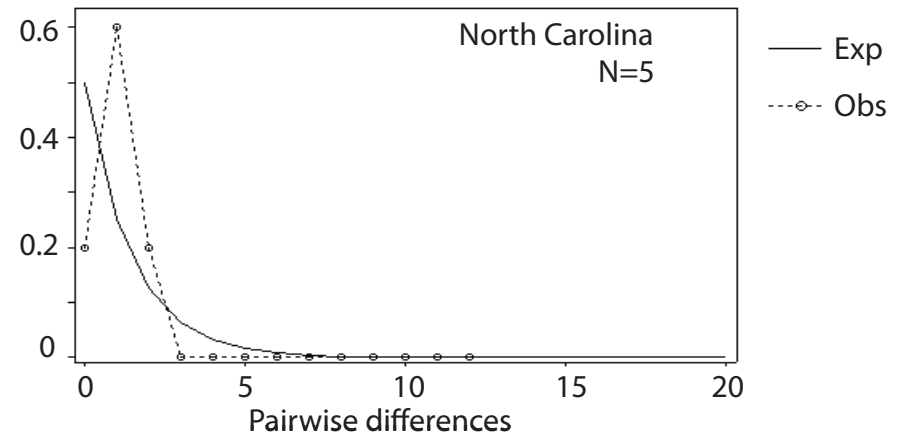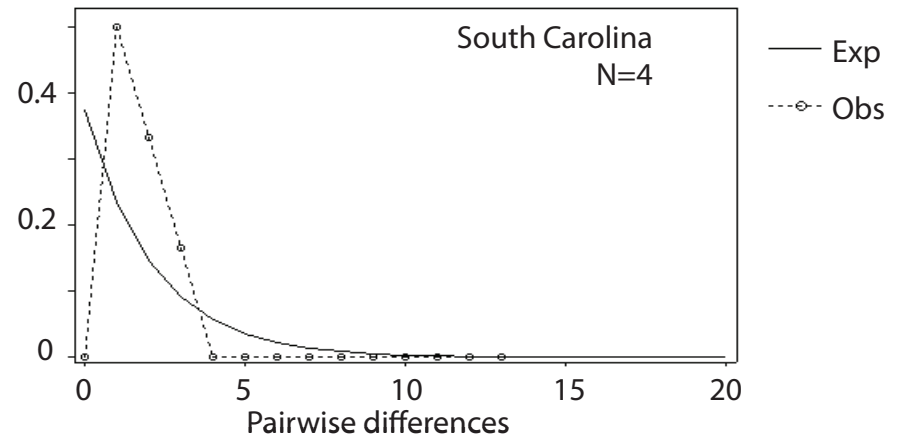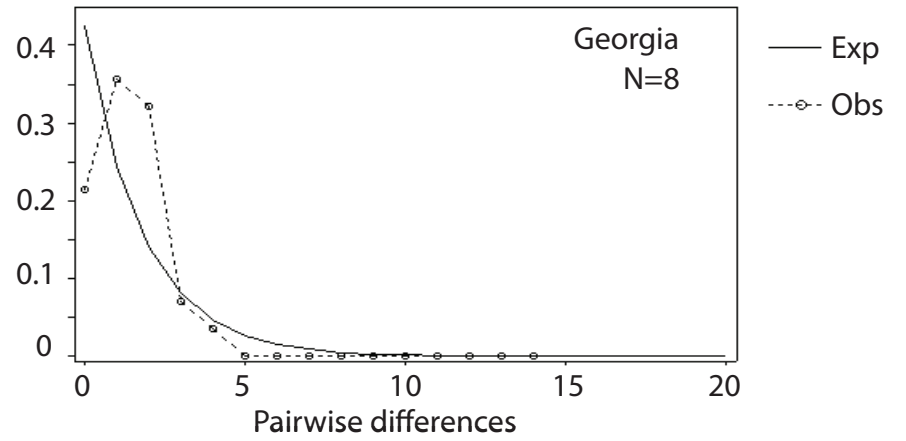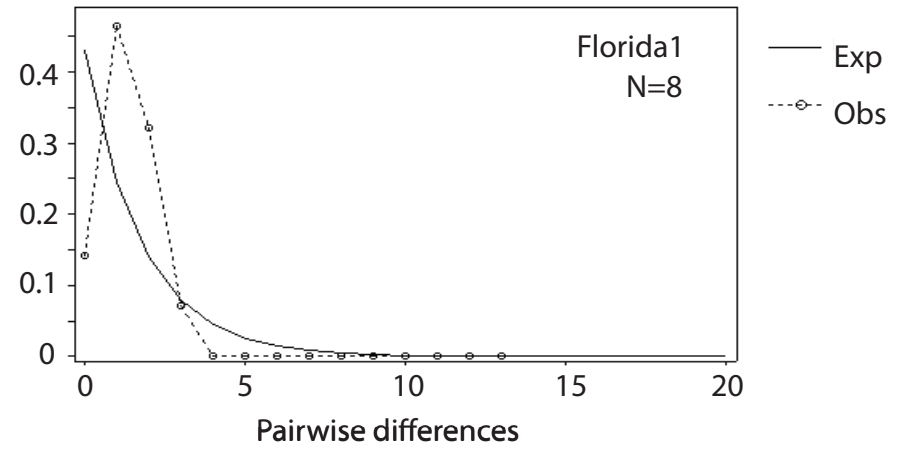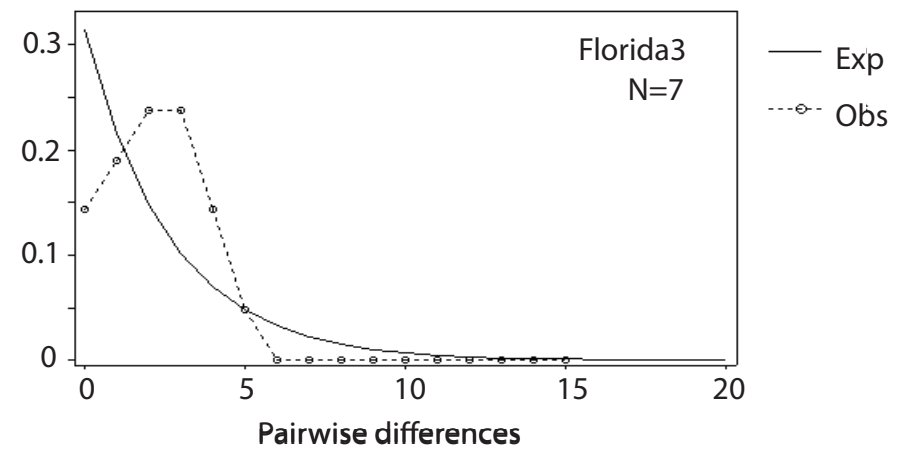

Supplement: Supplementary file 2 — Supplementary material 2 (PDF 297 kb) [file 227_2017_3132_MOESM2_ESM.pdf]
